# Dibutyl phthalate exposure induces thyroid toxicity through follicular cell pyroptosis *via* the NRF2/KEAP1/NF-κB pathway

**DOI:** 10.1080/07853890.2026.2643496

**Published:** 2026-03-16

**Authors:** Jieyi Wang, Fangda Fu, Yuying Chen, Keqi Fu, Huan Luo, Jiong Yang, Hongfeng Ruan

**Affiliations:** a Institute of Orthopaedics and Traumatology, The First Affiliated Hospital of Zhejiang Chinese Medical University (Zhejiang Provincial Hospital of Chinese Medicine), Hangzhou, China; b Department of General Surgery, Sir Run Run Shaw Hospital, Zhejiang University School of Medicine, Hangzhou, China; c The Fourth Clinical Medical College, Zhejiang Chinese Medical University, Hangzhou, China; d Department of Physical Education, Zhejiang Chinese Medical University, Hangzhou, China; e Department of Pharmacy, The Second Affiliated Hospital, School of Medicine, Zhejiang University, Hangzhou, China; f Hangzhou Fuyang Hospital of TCM Orthopedics and Traumatology, Hangzhou, Zhejiang, China

**Keywords:** Dibutyl phthalate, thyroid gland, pyroptosis, oxidative stress, NRF2-KEAP1 pathway

## Abstract

**Background:**

Dibutyl phthalate (DBP) is a plasticizer that bioaccumulates in organisms through multiple exposure routes. Although previous studies have documented DBP’s detrimental effects on the reproductive tract, liver, and neurodevelopment, the mechanisms underlying DBP-induced thyrotoxicity are inadequately understood.

**Objectives:**

To determine whether subchronic DBP exposure induces thyrotoxicity progression *via* thyroid follicular cell pyroptosis mediated by the NRF2/KEAP1/NF-κB pathway.

**Methods:**

Four-week-old male C57BL/6 mice were exposed to 50 or 250 mg/kg DBP by gavage five times weekly for 8 weeks. Systemic toxicity was assessed through body weight measurements and serum oxidative stress markers. Thyroid endocrine function and follicular morphology were evaluated *via* histopathological analysis. The molecular pathways regarding thyrotoxicity were determined using immunofluorescence analysis.

**Results:**

DBP exposure induced systemic toxicity, as evidenced by reduced body weight and elevated serum oxidative stress markers. Thyroid dysfunction was observed, including disrupted endocrine function and altered follicular morphology, accompanied by increased apoptosis, macrophage infiltration, and excessive inflammatory cytokine production. Notably, DBP promoted pyroptosis in thyroid follicular cells, as indicated by upregulated expression of NLRP3, ASC, CASPASE-1, and GSDMD. Mechanistically, DBP suppressed the NRF2/KEAP1 antioxidative pathway while activating NF-κB signalling.

**Conclusions:**

DBP induces thyrotoxicity through oxidative stress, inflammation, and pyroptosis, mediated by NRF2/KEAP1 suppression and NF-κB activation. These results provide novel insights into the mechanisms of DBP-induced thyroid damage and highlight potential health risks associated with prolonged exposure.

## Introduction

Dibutyl phthalate (DBP) is a phthalic acid ester (PAE) widely used as a plasticizer in medical devices, food packaging, and personal care products [1]. Due to its widespread use, DBP readily leaches from these products and bioaccumulates in organisms through multiple exposure routes, including inhalation, ingestion, and dermal contact [2]. This ubiquitous exposure raises significant concerns about its potential impact on human health, particularly as an endocrine disruptor [2]. Previous studies on environmental PAE have documented disturbance of thyroid function following subchronic exposure [3–5]. For example, di-(2-ethylhexyl) phthalate (DEHP) disrupts thyroid hormone and homeostasis and the hypothalamic-pituitary-thyroid (HPT) axis by affecting thyroid hormone biosynthesis, secretion, transportation, and metabolism [6,7]. Recent toxicological evidence has demonstrated that DBP exposure alters serum thyroid hormone levels [8,9]. Given these findings and the structural similarities between DBP and DEHP, DBP may pose comparable thyrotoxicity risks. Furthermore, numerous studies have demonstrated DBP’s detrimental effects on various organ systems, including the reproductive tract [10], liver [11], and neurodevelopment [12]. However, its specific impact on the thyroid gland, a crucial endocrine organ responsible for regulating metabolism and development, remains inadequately understood.

The thyroid gland is composed of spherical thyroid follicles surrounded by follicular cells, secretes thyroxine (T_4_) and triiodothyronine (T_3_) to regulate development and metabolism [13]. The pituitary hormone thyrotropin (TSH) regulates the synthesis and secretion of thyroid hormones, as well as thyrocyte proliferation, differentiation, and function [14], while T_3_ and T_4_ provide negative feedback to TSH *via* the hypothalamic-pituitary-thyroid (HPT) axis [15]. Accumulating evidence indicates that DBP has been detected in human metabolites, and population-based cohort studies suggest that DBP exposure alters thyroid hormone levels, including free and total T_4_ (fT_4_ and TT_4_), and free and total T_3_ (fT_3_ and TT_3_), and TSH [8,16], potentially contributing to thyroid disorders [17,18]. A recent study in Sprague–Dawley rats reported that subacute DBP exposure (50, 250, and 500 mg/kg) for two weeks led to a dose-dependent increase in TSH and T_3_ but a decrease in T_4_, and impaired thyroid structure [8]. Despite these observations, the underlying mechanisms of DBP-induced thyrotoxicity remain incompletely elucidated.

Pyroptosis is a form of proinflammatory programmed cell death triggered by the Nod-like receptor protein-3 (NLRP3)/Apoptosis-associated spot-like protein (ASC)/CASPASE-1 complex. This complex mediates the cleavage of gasdermin D (GSDMD) to generate the GSDMD-NT fragment, which forms membrane pores [19]. Concurrently, the maturation and substantial release of IL-1β and IL-18 occur, thereby amplifying the inflammatory response [19]. Growing evidence reveals the crucial involvement of thyroid follicular cell pyroptosis in the pathogenesis of thyrotoxicity, leading to destruction of thyroid structure and impairment of endocrine function [8,20]. Moreover, DBP exposure, alone or in combination with other contaminants, contributes to pyroptosis in various cells, such as renal tubular epithelial cells, cardiac cells, spleen macrophages, and liver cells, thereby aggravating damage to the kidneys, heart, and liver [11,21,22]. This evidence suggests that thyroid follicular cell pyroptosis may also play a critical role in DBP-induced thyroid dysfunction.

The nuclear factor E2-related factor 2 (NRF2)/Kelch-like ECH-associated protein 1 (KEAP1) pathway is a key regulator of oxidative stress and cellular homeostasis: in response to oxidative stress, NRF2 dissociates from KEAP1, translocates to the nucleus, and activates the transcription of antioxidant genes such as *heme oxygenase 1* (*HO-1*), thereby enhancing the cell’s ability to resist exogenous stress and reducing cellular damage [23]. Activation of this pathway has been implicated in maintaining thyroid homeostasis [24]. Our recent findings demonstrate that inhibition of the NRF2/KEAP1 pathway mediates NF-κB signaling induced thyroid follicular cell pyroptosis [20], while other studies have indicated that DBP exposure causes oxidative stress and injury in the thyroid and testis by inhibiting the NRF2/KEAP1 pathway, as indicated by decreased expression of NRF2 and its downstream target gene, HO-1 [24,25]. We therefore hypothesize that DBP exposure induces thyroid toxicity through inhibition of the NRF2/KEAP1 pathway, leading to NF-κB activation and thyroid follicular cell pyroptosis.

Given the widespread exposure to DBP and its documented toxicity, we aimed to comprehensively assess the impact and underlying mechanism of subchronic DBP-induced thyrotoxicity. Four-week-old male C57BJL/6J mice were exposed to DBP (0, 50, or 250 mg/kg) by gavage five times weekly for 8 weeks. Body weight, systemic oxidative stress status, thyroid structure and endocrine function, and underlying molecular alterations were evaluated using ELISA, histological staining, immunohistochemistry analysis, and TUNEL assay. Our findings offer novel insights into the subchronic thyrotoxicity induced by DBP.

## Materials and methods

### Reagents and antibodies

DBP was sourced from Sigma Aldrich (St. Louis, MO, USA). Assay kits for malondialdehyde (MDA) (A003-1-2), total antioxidant (T-AOC) capacity (A015-1-2), IL-1β (H002), fT_3_ (H222), fT_4_ (H223), and TSH (H087-1-2) were acquired from Nanjing Jiancheng Bioengineering Institute (Nanjing, China). The Periodic Acid-Schiff (PAS) staining kit was bought from Solarbio (Beijing, China). TUNEL bright green apoptosis detection kit was purchased from Vazyme Biotech Co. (Nanjing, China). Vector True VIEW Autofluorescence Quenching Kit (SP-8500-15) was from Vector Laboratories Inc (Newark, NJ, USA). Primary antibodies against BAX, BCL-2, BCL-X, TNF-α, IL-18, and IL-1β were from Ruiying Biological Co. (Jiangsu, China). Primary antibodies against F4/80, NLRP3, CASPASE-1, and HO-1 were purchased from Proteintech (Wuhan, China). Primary antibody against IL-6 was obtained from HuaBio (Hangzhou, China). Primary antibody against ASC was provided by Bioss Antibodies (Beijing, China). Antibody against GSDMD was supplied from Abcam (Cambridge, MA, USA). Primary antibodies against NRF2 and KEAP1 were supplied by ImmunoWay (Plano, TX, USA), while primary antibodies against NF-κB (p65) and phospho-NF-κB p65 (Ser536) (p-p65) were from Cell Signaling Technology (Beverly, MA, USA). Fluorescent secondary antibody was provided by Sungene Biotech Co. (Tianjin, China). Unless otherwise specified, all remaining reagents were from Sigma-Aldrich.

### Animals and treatment

Thirty 4-week-old male C57BL/6J mice were supplied by the Animal Experiment Center of Zhejiang Chinese Medical University (Grade SPF, SCXK (Shanghai): 2017-0005). All mice were housed in a well-ventilated, pathogen-free facility at a constant temperature of 23 ± 2 °C and relative humidity of 50%, maintained on a standard laboratory diet and water *ad libitum*, under a 12-hour light/dark cycle. All ethical regulations and protocols were strictly followed in accordance with the Ethics Committee on Animal Experimentation of Zhejiang Chinese Medical University (NO. 20220103-02) and were conducted in accordance with the ARRIVE Guidelines (Animal Research: Reporting of *In Vivo* Experiments) and the 3Rs principles (Replacement, Reduction, and Refinement) for laboratory animal welfare.

The mice were randomly divided into three groups: Vehicle group, Low-dose DBP group, and High-dose DBP group (*n* = 10 each). Animals were randomized into cages. Cages were randomly placed on racks. The doses (50 and 250 mg/kg) were selected based on previous DBP toxicology studies [8,12] and represent doses that do not exceed 1/32 of the LD_50_ in mice [26]. The 8-week duration was chosen to assess subchronic cumulative effects on thyroid function. These groups were administered 0, 50, or 250 mg/kg DBP dissolved in equal volumes of corn oil (five times per week) for 8 weeks. After the treatment period and a 24-hour fast, all mice were anesthetized with 2% sodium pentobarbital (40 mg/kg, *i.p.*) to ensure unconsciousness and minimize pain during tissue collection. Serum and thyroid tissues were collected for subsequent analysis, as previously described [20]. All experimental animals completed treatment and subsequent sample collection, with no deaths, health deterioration, or data exclusion from the analysis during the study. Additionally, no animals regained consciousness during the euthanasia process (conducted *via* an overdose of 2% sodium pentobarbital following AVMA guidelines). All animal-related procedures were supervised by the professional animal care staff of Zhejiang Chinese Medical University to ensure compliance with ethical standards. The order of measurements was randomized across groups to prevent systematic bias. The investigators performing the outcome assessment and data analysis were blinded to the group allocation.

### Serum biomarker assays

Thyroid hormones (T_4_ and T_3_) bind extensively to plasma proteins (> 99.7%), with only a small fraction in an unbound (free) form (fT_4_ and fT_3_) [27]. The unbound forms are biologically active [28]. Variations in TSH, fT_4_, and fT_3_ levels serve as reliable markers for thyroid function alterations. Accordingly, serum levels of fT_3_, fT_4_, TSH, and IL-1β were determined using specific ELISA kits following the manufacturers’ instructions. Serum MDA and T-AOC capacities were analyzed using commercial kits.

### Histopathological analysis

Thyroid tissues were fixed in 4% paraformaldehyde, dehydrated, embedded with OCT (Sakura Finetek, Japan), and sectioned at 8 μm thickness. Thyroid morphological alterations were evaluated using haematoxylin–eosin (HE) staining and PAS staining. Images were captured with a light microscopy (Carl Zeiss, Göttingen, Germany) at 10 × and 20 × magnifications. The diameter of the thyroid follicle was calculated by averaging the lengths of four straight lines drawn through the center of the follicle in four directions (0°, 45°, 90°, 135°). Six follicles in four randomly selected visual fields per section were analysed. The number of follicles was determined by counting those within a fixed field, including only those crossing the left and upper margins if they intersected the borders. Additionally, the thickness of follicular epithelial cells was assessed by measuring six randomly selected follicles, and the results were expressed in micrometres.

### Immunofluorescence (IF) analysis

The sections were blocked and incubated with primary antibodies at 4 °C overnight, including BCL-2 (1:400), BAX (1:400), BCL-X(1:400), CASPASE-3 (1:400), F4/80 (1:400), IL-6 (1:300), TNF-α (1:400), IL-18 (1:300), IL-1β (1:300), NLRP3 (1:300), ASC (1:300), CASPASE-1 (1:300), GSDMD (1:300), NRF2 (1:400), KEAP1 (1:400), HO-1 (1:400), p-p65 (1:400), and p65 (1:400). Fluorescent-conjugated secondary antibody was incubated for 1 h in the dark and counterstained with mounting medium (containing DAPI) (Solarbio Science & Technology, Beijing, China). No specific immunofluorescence signals were detected in negative control sections, confirming the specificity of the staining. Additionally, these antibodies have been previously validated in mouse thyroid tissue in our published studies [20]. Images were captured using a fluorescence microscope (Zeiss, Göttingen, Germany). The integrated optical densities or the number of positive-staining cells in the regions of interest were evaluated using ImageJ software (National Institutes of Health, Bethesda, MD, USA).

### TUNEL assay

TUNEL staining, which detects apoptosis by visualizing nuclear DNA fragmentation, was carried out in accordance with the manufacturer’s instructions. Sections were treated with proteinase K (1:100) for 20 min, equilibrated with equilibration buffer (1:4) for 30 min, incubated with Terminal Deoxynucleotidyl Transferase (TdT)-mediated reaction mixture, and finally mounted with antifading mounting medium (containing DAPI). A negative control was established using PBS instead of the TdT enzyme solution. Positive cells were counted in six randomly selected fields of view in three samples.

### Statistical analysis

All numerical data are expressed as mean ± SD. The normality of the data distribution was verified using the Shapiro-Wilk test. Comparisons among groups were performed using one-way ANOVA followed by Tukey’s multiple comparison test, using GraphPad Prism 8.0 software (GraphPad Software Inc., La Jolla, CA, USA). A *P*-value of < 0.05 was considered statistically significant.

## Results

### Subchronic DBP exposure induces systemic toxicity

**Figure 1.**
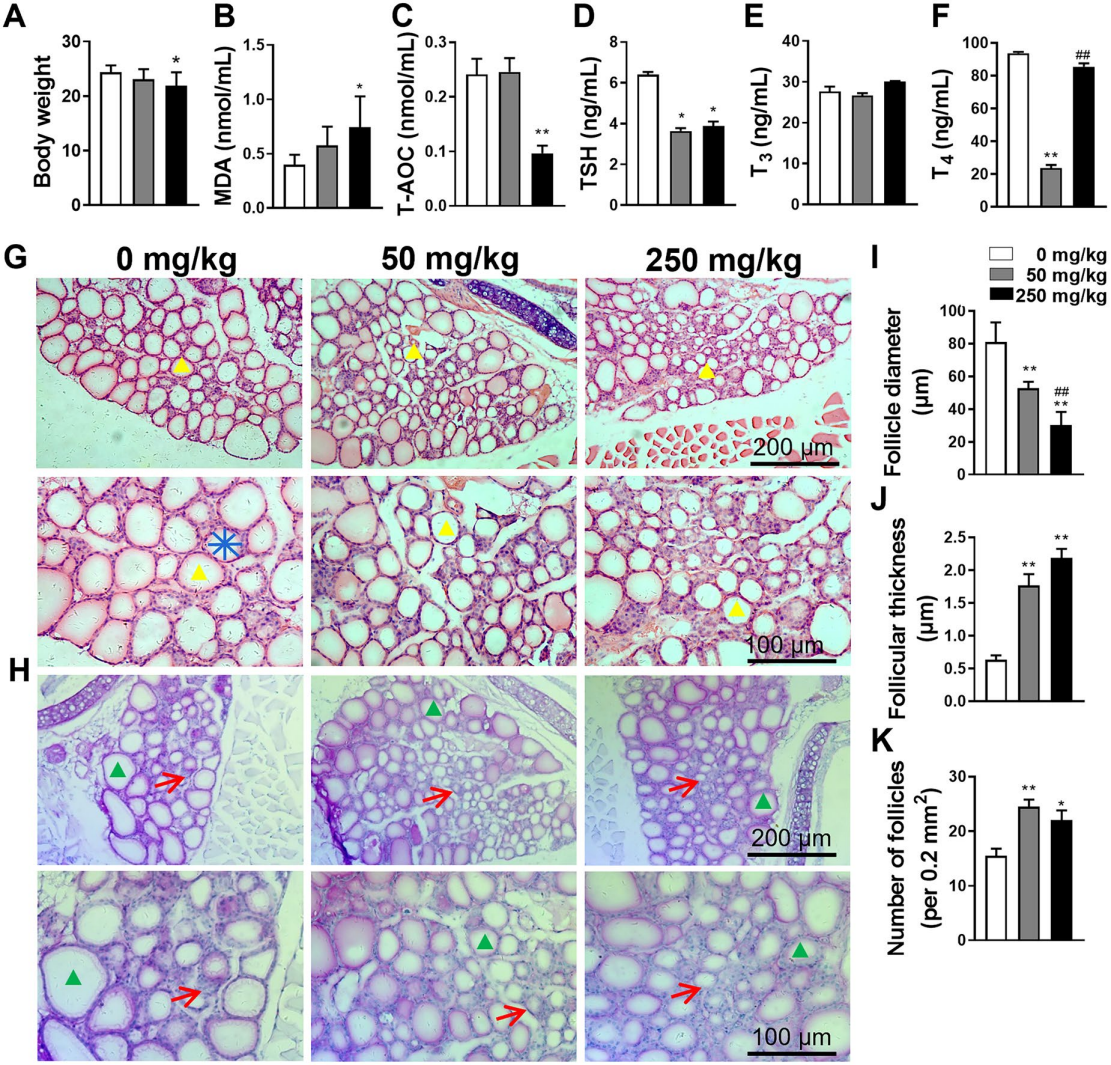
Effects of DBP exposure on body weight, serum biomarkers, and thyroid histopathology in mice. (A) The body weight of mice was determined after 8 weeks of DBP exposure. (B–C) Serum MDA and T-AOC capacities were examined using commercial kits. (D–F) Serum fT_3_, fT_4_, and TSH levels were evaluated by ELISA. (G–H) Thyroid histology was assessed by HE staining (G) and PAS staining (H). Yellow arrowheads indicate thyroid follicles, green arrowheads represent thyroid follicles, and red arrows denote follicular epithelial cells. Blue lines show follicle diameter. (I) Quantification of follicle diameter. The diameter was calculated by averaging the lengths of four straight lines drawn through the follicle centre at 0°, 45°, 90°, 135°. Six follicles in four randomly selected visual fields per group were analysed. (J–K) Quantification of follicle number and follicular epithelial cell thickness. Data are expressed as Mean ± SD (*n* = 5). *p < 0.05, **p < 0.01 (*vs.* Vehicle group); ^##^*p* < 0.01 (*vs.* 50 mg/kg DBP group).

To understand the toxic effects of DBP on the thyroid, we administered 50 or 250 mg/kg DBP to 4-week-old male mice over 8 weeks to assess subchronic toxicity. No expected or unexpected adverse events occurred during the experiment. A previous study has identified a negative association between PAE exposure and body mass index z-score in children [29], indicating the potential detrimental effects of PAE on normal development. Therefore, we first assessed body weight changes and found a dose-dependent reduction following DBP exposure, although the 50 mg/kg DBP group did not differ significantly from the Vehicle group (Figure 1(A)). To evaluate the impact of DBP on overall oxidative stress, we measured serum levels of MDA and T-AOC, indicators of oxidative stress. The 250 mg/kg DBP group showed a marked increase in MDA alongside a reduction in T-AOC, whereas the 50 mg/kg DBP group exhibited a non-significant increase in MDA and no significant change in T-AOC (Figure 1(B–C)). These findings suggest that subchronic DBP exposure reduces body weight and dose-dependently induces oxidative stress in mice. To determine thyroid-specific toxicity, we subsequently evaluated thyroid hormone levels, tissue structure, and molecular alterations in thyroid follicular cells.

### Subchronic DBP exposure disrupts thyroid hormone homeostasis

To explore whether DBP affects thyroid function, we analysed serum levels of TSH, fT_3_, and fT_4_ using ELISA. The DBP-treated groups exhibited notably lower levels of TSH and fT_4_, while fT_3_ levels remained unchanged, indicating impaired thyroid endocrine function (Figure 1(D–F)). Specifically, DBP at 50 and 250 mg/kg reduced TSH levels to 57% and 61% of the Vehicle group, respectively, while fT_4_ levels decreased by 75% and 9% (Figure 1(D,F)).

### Subchronic DBP exposure damages thyroid structure

Given the observed impairments in DBP-induced thyroid function, it is crucial to determine whether these effects are mediated by structural alterations in the thyroid. Histological analysis using HE staining and PAS staining revealed notable disruptions in the typical follicular architecture in thyroids from DBP-treated mice compared to Vehicle-treated mice (Figure 1(G,H)). Notably, there was significant thickening of interstitial tissue and infiltration of inflammatory cells, indicating an inflammatory response to DBP exposure. Treatment with 50 and 250 mg/kg DBP caused a dose-dependent reduction in thyroid follicle diameter and increased the thickness of thyroid follicular epithelial cells (Figure 1(I–J)). Additionally, DBP exposure significantly increased the number of follicles, with the 50 mg/kg group exhibiting slightly more follicles than the 250 mg/kg group (Figure 1(K)). These findings demonstrate that DBP disrupts thyroid structure, potentially contributing to its detrimental effects on thyroid function.

### Subchronic DBP exposure amplifies thyroid apoptosis

**Figure 2.**
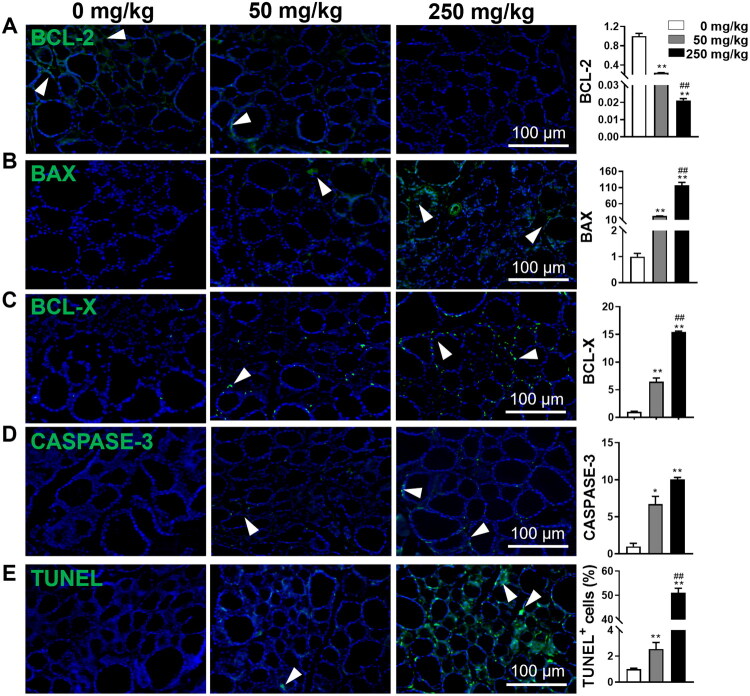
DBP exposure facilitates apoptosis in thyroid tissues. (A–D) IF staining and quantitative analysis of thyroid tissues for BCL-2 (A), BAX (B), BCL-X (C), and CASPASE-3 (D). (E) TUNEL staining and quantitative analysis of thyroid tissues to visualize DNA fragmentation. DAPI was utilized for nuclei staining (blue). Arrowheads indicate positive staining in the respective panels. Data are expressed as Mean ± SD (*n* = 5). *p < 0.05, **p < 0.01 (*vs.* Vehicle group); ^##^*p* < 0.01 (*vs.* 50 mg/kg DBP group).

To determine whether subchronic DBP exposure affects thyroid apoptosis, we analyzed the expression of apoptosis-related proteins, BCL-2, BAX, BCL-X, and CASPASE-3, using IF analysis. Consistent with the above findings, DBP dose-dependently potentiated apoptosis, as shown by the upregulation of BAX, BCL-X, and CASPASE-3, along with the downregulation of BCL-2 (Figure 2(A–D)). TUNEL staining further confirmed these results, showing that 50 and 250 mg/kg DBP increased TUNEL-positive cells by 2.5-fold and 51-fold compared to the Vehicle group, respectively (Figure 2(E)). These findings indicate that DBP exposure significantly elevates apoptosis in thyroid tissue.

### Subchronic DBP exposure facilitates inflammatory response in thyroid tissue

**Figure 3.**
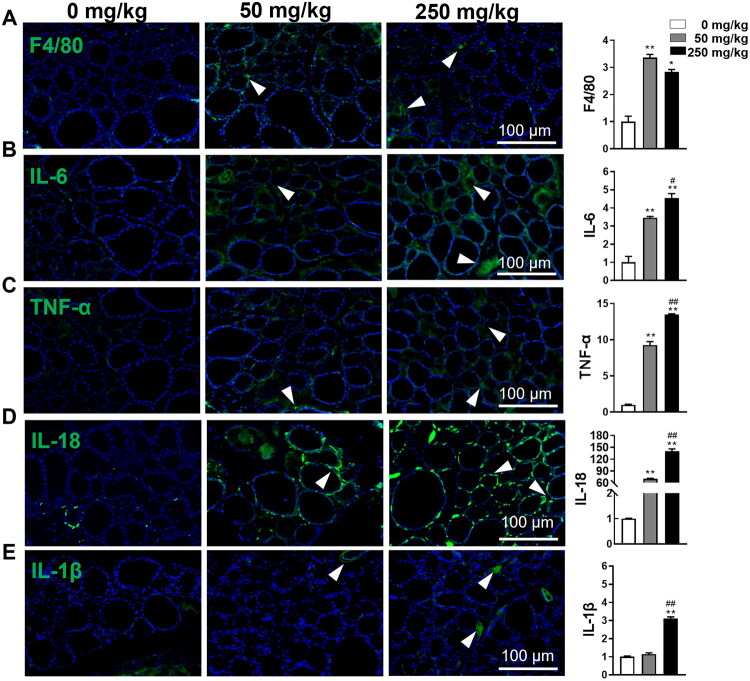
DBP promotes macrophage infiltration and increases inflammatory cytokines in the thyroid of mice. (A) IF staining and quantitative analysis for the expression of F4/80 in thyroid tissues. (B–E) IF staining and quantification for the expression of inflammatory cytokines, IL-6 (B), TNF-α (C), IL-18 (D), and IL-1β (E) in the thyroid. Arrowheads indicate positive staining in the respective panels. Data are expressed as Mean ± SD (*n* = 5). *p < 0.05, **p < 0.01 (*vs.* Vehicle group); ^#^*p* < 0.05, ^##^*p* < 0.01 (*vs.* 50 mg/kg DBP group).

Considering the substantial cell infiltration observed in DBP-treated mice thyroid tissue on HE staining, we further investigated the inflammatory response by quantifying macrophage infiltration and inflammatory cytokine expression in thyroid tissue. IF analysis of F4/80, a macrophage-specific marker, revealed a significant increase in F4/80-positive cells in the thyroid tissue of DBP-treated mice, with no marked difference between the 50 and 250 mg/kg DBP groups (Figure 3(A)). Furthermore, IF analysis of inflammatory cytokines, including IL-1β, IL-18, IL-6, and TNF-α, revealed a dose-dependent increase in their levels, except for IL-1β, which showed no significant upregulation at 50 mg/kg (Figure 3(B–E)). Notably, 250 mg/kg DBP exposure led to a dramatic 140.6-fold increase in IL-18 levels, making it the most significantly altered cytokine. Additionally, ELISA analysis of serum IL-1β confirmed that neither 50 nor 250 mg/kg DBP exposure significantly affected its levels (data not shown). Based on the above results, we hypothesized that DBP exposure contributes to inflammatory cell infiltration and response in thyroid tissues, with IL-18 serving as a key mediator.

### DBP triggers thyroid follicular cell pyroptosis

**Figure 4.**
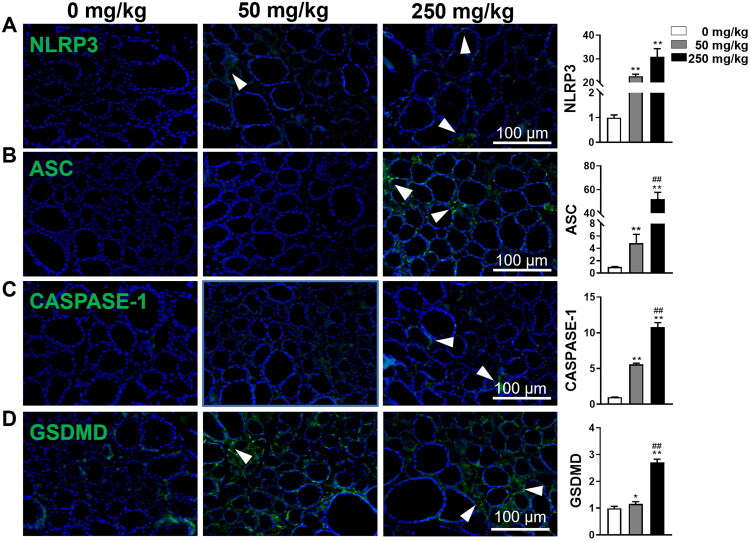
DBP exposure activates pyroptosis in thyroid follicular cells of mice. (A–D) IF staining and quantification for the expression of NLRP3 (A), ASC (B), CASPASE-1 (C), and GSDMD (D) in the thyroid after 8 weeks of DBP exposure. Arrowheads indicate positive staining in the respective panels. Data are expressed as Mean ± SD (*n* = 5). *p < 0.05, **p < 0.01 (*vs.* Vehicle group); ^##^*p* < 0.01 (*vs.* 50 mg/kg DBP group).

Since DBP exposure stimulated inflammatory cytokine production, particularly IL-18, in the thyroid tissue, we subsequently evaluated pyroptosis activity by examining pyroptosis-related proteins, including NLRP3, ASC, CASPASE1, and GSDMD. IF analysis showed elevated expression of these proteins in thyroid tissues of DBP-treated mice, with the most significant enhancement observed in the 250 mg/kg group (Figure 4(A–D)). Notably, exposure to 50 mg/kg DBP resulted in a 22.7-fold increase in NLRP3 and a 4.8-fold increase in ASC levels, while 250 mg/kg DBP treatment led to a 30.8-fold increase in NLRP3 and an impressive 52.1-fold increase in ASC compared to the vehicle group (Figure 4(A–B)). These results suggest that DBP induces excessive production of inflammatory factors primarily through the activation of NLRP3-mediated pyroptosis in thyroid follicular cells.

### DBP regulates NRF2/KEAP1 and NF-κB pathways in the thyroid

**Figure 5.**
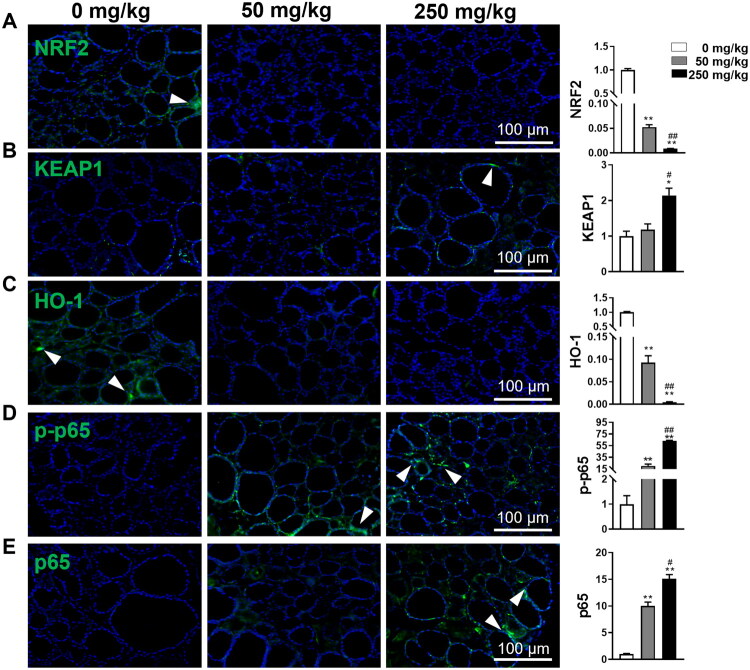
DBP suppresses the NRF2/KEAP1 pathway and activates the NF-κB pathway in thyroid tissues. (A–C) IF staining and quantification for NRF2 (A), KEAP1 (B), and HO-1 (C) in thyroid tissues. (D-E) IF staining and quantification for p-p65 (D) and total p65 (E) protein expression. Arrowheads indicate positive staining in the respective panels. Data are expressed as Mean ± SD (*n* = 5). *p < 0.05, **p < 0.01 (*vs.* Vehicle group); ^#^*p* < 0.05, ^##^*p* < 0.01 (*vs.* 50 mg/kg DBP group).

To elucidate the mechanism by which DBP stimulates pyroptosis in thyroid follicular cells, we examined the NRF2/KEAP1 and NF-κB pathways by analysing the expression levels of NRF2, KEAP1, and HO-1, as well as the phosphorylation status of p65, a crucial component of NF-κB signalling. As shown in Figure 5(A–E), DBP significantly increased the expression of KEAP1, p-p65, and total p65, while reducing NRF2 and HO-1 levels in thyroid tissue, except for a non-significant increase in KEAP1 in the 50 mg/kg group. Taken together, these results suggest that DBP triggers oxidative stress and promotes pyroptosis in thyroid follicular cells through the suppression of the NRF2/KEAP1 pathway and activation of the NF-κB pathway, thereby exacerbating thyroid toxicity.

## Discussion

**Figure 6.**
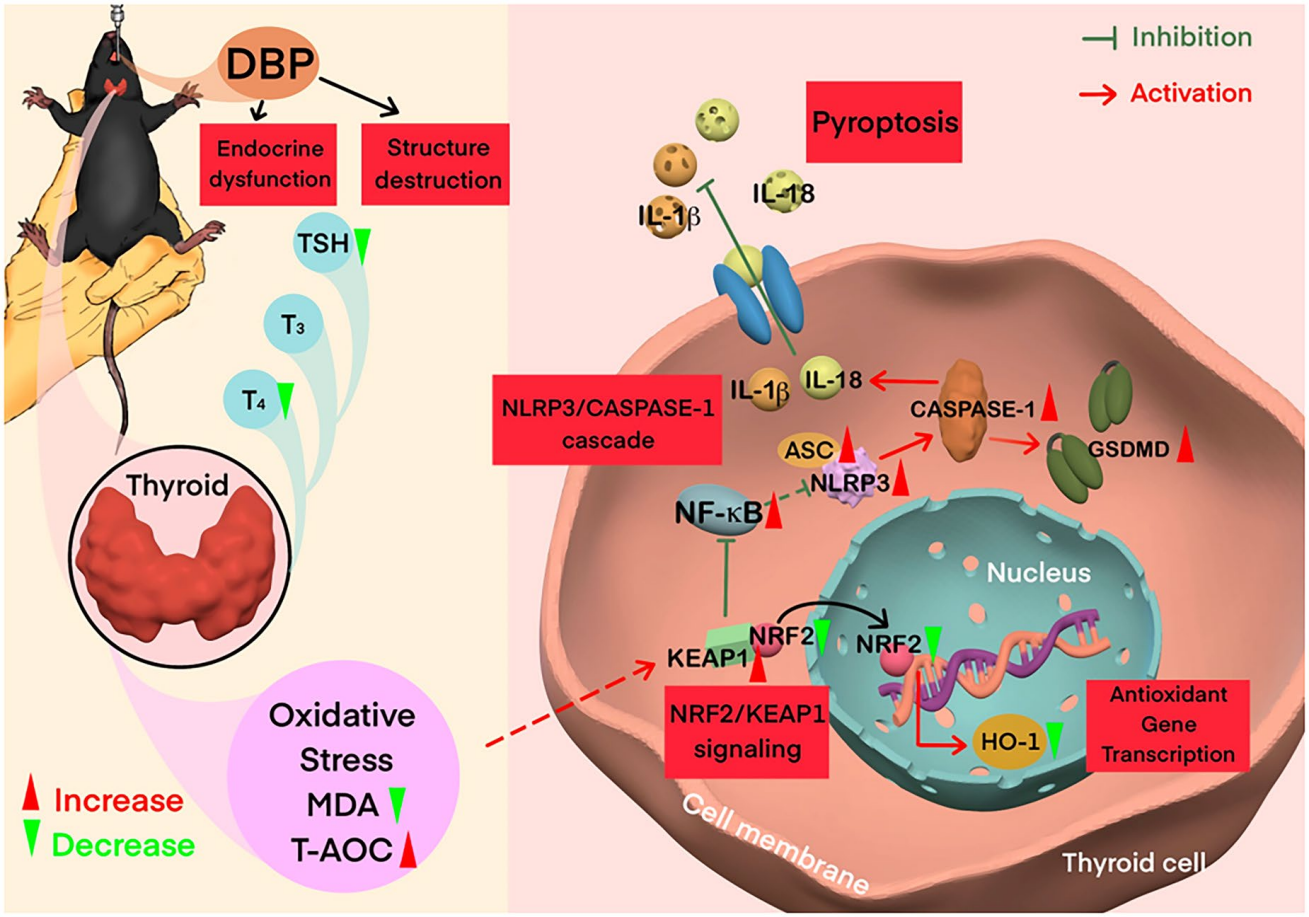
Schematic working model of DBP-induced thyrotoxicity. Schematic representation illustrating how DBP induces thyrotoxicity by suppressing the NRF2/KEAP1 pathway and promoting thyroid follicular cell pyroptosis.

DBP-induced thyroid toxicity has been documented in multiple studies; however, the cellular and molecular mechanisms underlying this toxicity remain incompletely understood. Our study provides novel mechanistic insights by revealing that DBP-induced thyrotoxicity is likely primarily driven by thyroid follicular cell pyroptosis, rather than apoptosis, mediated through the NRF2/KEAP1/NF-κB signalling axis. Specifically, our findings reveal a mechanistic cascade whereby DBP exposure suppresses the NRF2/KEAP1 antioxidant defence system, leading to oxidative stress and subsequent activation of NF-κB signalling. This NF-κB activation then triggers NLRP3 inflammasome assembly and pyroptotic cell death, characterized by dramatic IL-18 release and extensive inflammatory cell infiltration (Figure 6). This integrated mechanistic framework extends beyond previous reports by identifying the specific signalling pathway linking oxidative stress to inflammatory cell death in DBP-induced thyrotoxicity, with important implications for understanding phthalate-induced endocrine disruption.

DBP exposure has been shown to impair various organs, suggesting a broad spectrum of effects [1]. Studies have frequently utilized DBP doses of 50, 250, 500, and 750 mg/kg to investigate its subacute and subchronic impacts in animal models, particularly in rats and mice [8,30–32]. For instance, subacute exposure to DBP (50, 250, or 500 mg/kg/day) in pregnant rodents accelerated puberty onset and testicular development in male offspring, resulting in reproductive system damage [33]. Moreover, DBP has been found to bioaccumulate in the thyroids of multiple species, including humans, rats, goldfish, Xenopus laevis, and zebrafish, leading to thyrotoxicity through adverse effects on the HPT axis and impairments in thyroid morphology and function [8,16, 34–36]. A recent study by Li et al. reported that subacute DBP exposure (50, 250, and 500 mg/kg/day for two weeks) in six-week-old male rats impaired thyroid structure and induced inflammation [8]. Based on these studies, we selected two doses (50 and 250 mg/kg/day), both no more than 1/32 of the LD_50_ value in mice [26], to investigate DBP’s subchronic toxicity while excluding the highest dose (500 mg/kg) to focus on the effects of long-term exposure to lower doses [8]. Moreover, while DBP has been designated as a priority pollutant by the U.S. Environmental Protection Agency (EPA), which has established a reference dose (RfD) for DBP of 0.1 mg/kg·day [37], our 8-week subchronic model is designed to mimic the cumulative effects of long-term human exposure. Additionally, Miura et al. demonstrated that *in vitro* glucuronidation rates of monobutyl phthalate (MBP) in mouse liver microsomes are slower than in human liver microsomes [38]. However, small rodents, including mice, have significantly higher basal metabolic rates (BMR) (approximately 7-fold higher) [39] and esterase activities compared to humans, while the primary hydrolysis step from DBP to MBP is rate-limiting in humans [40], resulting in slower overall *in vivo* clearance of DBP in humans than in mice [38]. Thus, higher doses were selected to compensate for the compressed experimental timeline and interspecies metabolic differences, thereby enabling meaningful mechanistic alterations. Furthermore, given DBP’s role as an environmental endocrine disruptor and its specific effects on male reproductive health [33], we used male mice as subjects in our experiment to avoid the confounding influence of its oestrogen-like effects.

T_4_ is synthesized and released by thyroid cells and is sensitive to changes in thyroid follicles, while T_3_ is mostly produced in the liver through extrathyroidal deiodination of T_4_, and TSH is secreted by the anterior pituitary in response to feedback from circulating levels of T_3_ and T_4_ [41]. Thyroid dysfunction is often indicated by imbalances of T_3_, T_4_, and/or TSH levels [41]. While subacute DBP exposure studies have consistently reported thyroid toxicity, results vary, leading to uncertainty regarding the underlying mechanisms. For instance, Li et al. observed that 50, 250, and 500 mg/kg DBP exposure in six-week-old male SD rats for two weeks led to a reduction in T_4_ levels, along with an increase in TSH and T_3_ [8]. In contrast, Majeed et al. reported a dose-dependent reduction in T_4_ levels without notable changes in T_3_ after exposing albino rats to 10, 50, and 100 mg/kg DBP for 4 weeks [2]. In the present study, we found that subchronic DBP exposure over 8 weeks decreased both TSH and fT_4_ levels, while fT_3_ levels remained largely unchanged, highlighting a disruption in thyroid hormone synthesis or regulatory mechanisms specific to TSH and T_4_. These variable patterns across studies underscore the complexity of thyroid endocrine responses to DBP, which appear to be influenced by exposure duration, dose, etc. Noteworthy, DBP exposure led to an approximately 40% reduction in TSH levels, and a striking 75% decrease in fT_4_ levels at 50 mg/kg, compared to a 9% decrease at 250 mg/kg. This paradoxical result may be attributed to the gradual and cumulative damage to the thyroid caused by the lower dose, leading to a more pronounced long-term effect, while the higher dose may induce early, severe damage, triggering compensatory repair mechanisms that stabilize T_4_ levels over time. Based on the above findings, we propose that short-term DBP exposure might broadly affect thyroid hormone levels (TSH, T_4_, and T_3_), while prolonged exposure of 4 weeks or more primarily disrupts TSH and T_4_ levels, potentially reducing thyroid responsiveness to stimuli or influencing hormone synthesis processes. Time-course studies examining hormone levels at multiple time points and assessment of thyroid regenerative markers would be needed to definitively establish the mechanisms underlying these dose-dependent differences.

Thyroid structural integrity is crucial for maintaining hormone homeostasis [42]. DBP exposure has been shown to induce thyroid apoptosis, subsequently compromising thyroid hormone synthesis and secretion [17], whereas inhibition of thyroid apoptosis can partially restore thyroid function [43]. Herein, our data demonstrated that DBP exposure elevated pro-apoptosis-related protein expression and TUNEL-positive cells, alongside reduced thyroid follicle diameters and increased epithelial cell thickness, indicating that DBP induces thyroid apoptosis, which likely contributes to the observed structural damage and may underlie the reduction of fT_4_ levels.

Inflammatory responses are critical to the pathogenesis of various thyroid diseases, characterized by macrophage infiltration, elevated production of inflammatory cytokines, and interstitial thickening [20,44]. Numerous toxicity studies have shown that DBP exposure can provoke aberrant inflammation responses in endocrine organs [45], including the thyroid [8]. Recent evidence points to a significant increase in F4/80-positive macrophage infiltration and excessive secretion of inflammatory cytokines, particularly IL-18, within thyroid tissues, contributing to tissue damage and endocrine dysfunction [20,46]. Our findings align with this, showing that subchronic DBP exposure resulted in marked macrophage infiltration and inflammatory cytokine production, and a sharp rise in IL-18 levels—up to 140.5 times higher than the Vehicle group. Unlike systemic inflammatory responses, this localized inflammation suggests that DBP directly damages thyroid tissues, exacerbating structural and functional impairments. Notably, pyroptosis, but not apoptosis, is characterized by the rapid release of pro-inflammatory factors [20]. Therefore, we propose that the thyrotoxicity observed within DBP-treated mice may be largely driven by pyroptosis-induced cytokine overproduction, rather than apoptosis alone.

Extensive investigations have shown that pyroptosis in thyroid follicular cells mediates inflammation and contributes to the progression of thyroid damage [8,20]. Studies have demonstrated that dysregulated activation of the NLRP3 inflammasome represents a crucial pathway underlying DBP-induced pyroptosis, leading to inflammation and metabolic disturbances [8,11,21]. Our study provides further evidence that DBP exposure upregulates the expression of pyroptosis-related proteins, including NLRP3, ASC, CASPASE1, and GSDMD, in thyroid tissues, with dose-dependent effects. Notably, a 30.8-fold increase in NLRP3 and a 52.1-fold increase in ASC expression were observed at 250 mg/kg dosage. To determine whether pyroptosis or apoptosis is the dominant mechanism driving DBP-induced thyroid toxicity, we compared the magnitude and functional consequences of these two cell death pathways. Multiple lines of evidence establish pyroptosis as the primary mechanism: First, pyroptosis markers exhibited substantially greater fold-changes (NLRP3: 30.8-fold, ASC: 52.1-fold) compared to apoptosis markers (TUNEL: 51-fold), with the pyroptosis-specific cytokine IL-18 showing a dramatic 140-fold increase. Second, the extensive macrophage infiltration and robust inflammatory cytokine production are hallmark features of pyroptosis, whereas apoptosis is generally immunologically silent. Third, these findings are consistent with our previous work demonstrating pyroptosis as the primary driver of thyroid toxicity progression [20]. Collectively, these results provide strong correlative evidence that pyroptosis appears to be a pivotal and likely dominant role in DBP-induced thyroid toxicity, surpassing the contribution of apoptosis. While our data strongly support this interpretation through multiple lines of evidence (magnitude of markers, inflammatory phenotype, and consistency with previous findings), mechanistic validation through pyroptosis inhibition or genetic ablation studies would be needed to definitively establish causality.

Oxidative stress profoundly affects thyroid physiology and is implicated in various thyroid disorders, including autoimmunity and cancer [47,48]. Emerging evidence highlights that dysregulation of the NRF2/KEAP1 pathway mediates aberrant oxidative stress, thereby influencing apoptosis, and pyroptosis, and inflammation [24,49,50]. Our latest research and that of others suggest that abnormalities in the NRF2/KEAP1 pathway are linked to NF-κB activation, which triggers thyroid follicular cell pyroptosis, contributing to thyroid dysfunction [20,51]. Moreover, DBP has been shown to induce oxidative stress and NF-κB/NLRP3-mediated pyroptosis in hepatocytes and renal tubular epithelial cells, thus promoting liver fibrosis and renal injury [11,21]. Consistent with these findings, our results demonstrate coordinated changes in pathway components: DBP exposure elevates KEAP1 and phosphorylated P65 levels while reducing NRF2 and HO-1 levels in follicular epithelial cells. These correlative findings suggest that DBP-mediated pyroptosis may exacerbate thyroid damage through suppressing the NRF2/KEAP1 antioxidant pathway and activating downstream NF-κB signaling. However, definitive establishment of causality would require intervention studies using pathway-specific modulators (e.g. NRF2 activators or NF-κB inhibitors) to validate the proposed NRF2/KEAP1/NF-κB/pyroptosis axis.

However, this study has several limitations. First, we did not measure DBP concentrations in serum and thyroid tissues, which limits a more comprehensive analysis of its cumulative effects on the body and thyroid. Meanwhile, although we utilized an 8-week subchronic exposure model, longer-term studies are needed to explore DBP’s persistent effects on thyroid function, including potential impacts on thyroid tumours and autoimmune diseases. Moreover, whether DBP-induced effects on body weight and thyroid toxicity can be reversed after DBP withdrawal remains unclear. Previous studies have shown inconsistent evidence regarding the association between PAEs (including DBP) exposure and thyroid diseases in humans [1]. Although animal models provide valuable insights, understanding the environmental and clinical relevance of DBP exposure in human populations is crucial. Epidemiological and preclinical studies should aim to clarify the health risks associated with DBP exposure levels and their correlation with thyroid toxicity. Such investigations will advance our understanding of DBP’s complex impact on thyroid function and structure, informing both environmental toxicology and public health strategies.

## Conclusion

To sum up, our findings demonstrate that DBP exposure disrupts antioxidant homeostasis and exerts thyrotoxicity by damaging thyroid structure and endocrine function. Mechanistically, DBP induces NLRP3-mediated pyroptosis of thyroid follicular cells and the subsequent release of inflammatory cytokines, likely mediated through inhibiting the NRF2/KEAP1 antioxidant pathway, thereby exacerbating thyroid toxicity. This study offers valuable insights into the molecular mechanisms underlying DBP-induced thyroid damage and highlights the potential health risks associated with prolonged exposure to DBP.

## Supplementary Material

Author_Checklist_Full.pdf

## Data Availability

The data supporting this study’s findings are available from the corresponding author, HFR, upon reasonable request.
